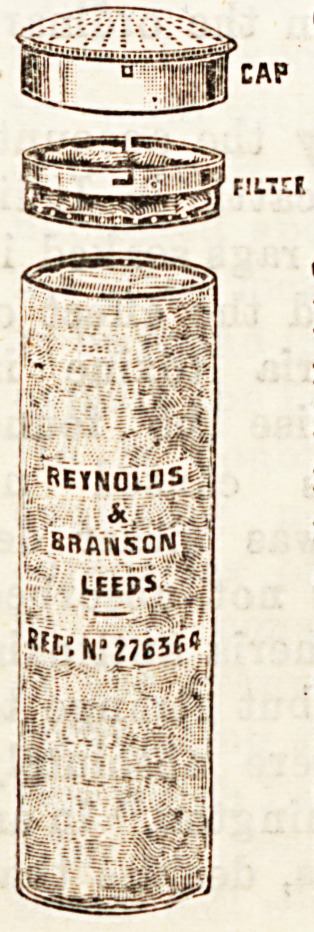# New Appliances and Things Medical

**Published:** 1896-09-12

**Authors:** 


### NEW APPLIANCES AND THINGS MEDICAL.

[We shall be glad to receive, at our Office, 428, Strand, London, W.C., from the manufacturers, specimens of all new preparations and appliances, which may be brought out from time to time.]

### CAFFYN'S MALTO-CARNIS.

(The Liquor Carnis Company, 192, Aldersgate Street, E.C.)

This concentrated food, prepared by the above company, consists of a combination of their well-known Liquor Carnis together with extract of malt and coca. Such a combination of food stuff and stimulants obviously renders the preparation a most valuable one, both from the standpoint of concentration and digestibility. For invalids, travellers, and cyclists it constitutes a useful standby, since a single dessert spoonful introduced into the stomach can be almost immediately absorbed into the system without taxing the powers of digestion, and yet provide as much pabulum for physiological energy as would ten times the bulk of ordinary mixed foods. It is decidedly pleasant to the palate, and can be taken in hot milk or otherwise diluted if the more concentrated syrup proves too strong.

### CELLULOID CYLINDER DRESSING-BOX.

(Reynolds and Branson, Leeds.)

Again we have the pleasant task of introducing to our readers a new invention of Messrs. Reynolds and Branson. The last novelty of this enterprising firm is a celluloid cylinder for surgical dressings intended for use in a doctor's bag. Not only is the material tough and light, but it also enables the possessor to see at a glance the nature of the dressing contained in the box, and the necessity of replenishing when it arises. It is fitted with an air filter in the cover after the system of the dust-proof drawers, introduced by the same firm. We regard the contrivance as an admirable one.

## Figures and Tables

**Figure f1:**